# Apochromatic X-ray focusing

**DOI:** 10.1038/s41377-023-01157-8

**Published:** 2023-05-04

**Authors:** Umut T. Sanli, Griffin Rodgers, Marie-Christine Zdora, Peng Qi, Jan Garrevoet, Ken Vidar Falch, Bert Müller, Christian David, Joan Vila-Comamala

**Affiliations:** 1 Paul Scherrer Institute, Laboratory for X-ray Nanoscience and Technologies, Forschungsstrasse 111, 5232 Villigen, Switzerland; 2 Biomaterials Science Center, Department of Biomedical Engineering, University of Basel, Hegenheimermattweg 167 B, 4123 Allschwil, Switzerland; 3 Deutsches Elektronen-Synchrotron DESY, Notkestr. 85, 22607 Hamburg, Germany

**Keywords:** Applied optics, Micro-optics, X-rays

## Abstract

Achromatic doublets are combinations of two individual lenses designed to focus different wavelengths of light in the same position. Apochromatic optics are improved versions of the achromatic schemes which extend the wavelength range significantly. Both achromatic and apochromatic optics are well-established for visible light. However, X-ray achromatic lenses did not exist until very recently, and X-ray apochromatic lenses have never been experimentally demonstrated. Here, we create an X-ray apochromatic lens system using an appropriate combination of a Fresnel zone plate and a diverging compound refractive lens with a tuned separation distance. The energy-dependent performance of this apochromat was characterized at photon energies between 6.5 and 13.0 keV by ptychographic reconstruction of the focal spot and scanning transmission X-ray microscopy of a resolution test sample. The apochromat delivered a reconstructed focal spot size of 940 × 740 nm^2^. The apochromatic combination shows a four-fold improvement in the chromatic aberration correction range compared to an achromatic doublet configuration. Thus, apochromatic X-ray optics have the potential to increase the focal spot intensity for a wide variety of X-ray applications.

## Abstract (web summary)

From achromatic to apochromatic X-ray lenses: a new chapter in X-ray microscopy. Our work demonstrates the first experimental realization of X-ray apochromatic lenses, paving the way for enhanced imaging and analysis capabilities in the field.

## Graphical abstract

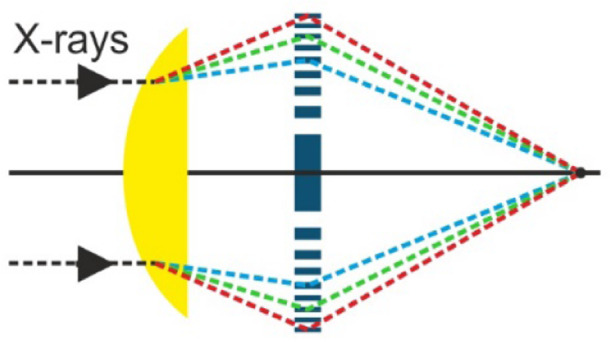

## Introduction

Diffractive and refractive lenses are widely used as focusing optics in high-resolution X-ray microscopy^1–6^. However, both are highly chromatic, meaning that they focus each wavelength to a different position along the optical axis. Consequently, X-ray imaging using chromatic lenses such as Fresnel zone plates (FZP) or compound refractive lenses (CRL) requires highly monochromatic light. Generally, monochromatization sacrifices a large portion of the X-ray intensity, though the exact fraction depends on the X-ray source and the monochromator. In the case of X-ray tube sources that typically generate low-brightness broadband radiation, X-ray microscopy setups suffer from a lack of X-ray intensity resulting in long acquisition times.

**Fig. 1:**
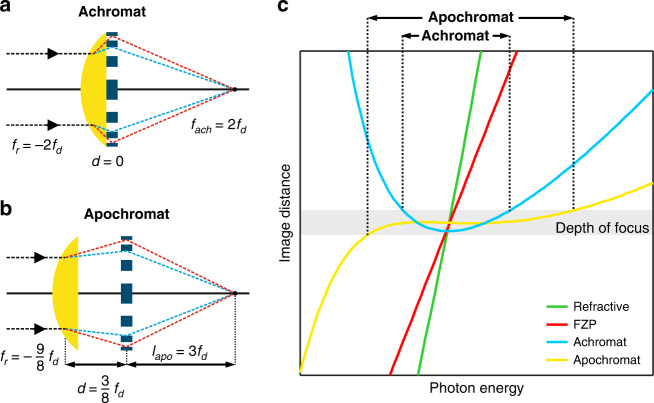
Geometrical considerations for the achromat and the apochromat. Conceptual design for (**a**) an X-ray achromat and (**b**) an X-ray apochromat by combining a refractive lens and a Fresnel zone plate. The resulting optical elements can be interpreted as a combination of two thin lenses. **c** Conceptual energy-dependent image distance curves (dispersion) are compared for an achromat and apochromat as well as a Fresnel zone plate and refractive lens. The ranges of the chromatic aberration correction for the achromat and apochromat are qualitatively shown on the top axis of the plot

A solution to the chromatic aberration for visible light lenses has existed for more than 100 years, for which achromatic doublets are constructed by combining two lenses made of glass with different dispersion and carefully selected curvatures. In the X-ray regime, approaches for an achromatic optic were theoretically proposed about two decades ago^7,8^. The most practical way to achieve an X-ray achromatic doublet is by combining a weakly diverging CRL with a strongly converging FZP in close contact (see Fig. 1a). The achromatic behavior originates from the differences in the dispersion of diffractive and refractive lenses, which are directly proportional to photon energy *E* (focal length *f*_d_ ∝ *E*) and to the square of *E* (focal length *f*_r_ ∝ *E*^2^), respectively. By selecting refractive and diffractive lenses with focal lengths so that *f*_r_ = −2*f*_d_ and bringing them into close contact (*d* = 0), an achromat is produced with a focal length of *f*_ach_ = 2*f*_d_, as depicted in Fig. 1a. An alternative configuration with *f*_r_ = −9*f*_d_/8 and a separation distance of *d* = 3*f*_d_/8 results in apochromatic focusing with an image distance *l*_apo_ = 3*f*_d_, as depicted in Fig. 1b. The apochromatic case offers a significantly improved chromatic aberration correction at the expense of requiring the production of refractive and diffractive elements with greater numerical apertures to achieve a comparable focal spot size. The achromatic case has dispersion dominated by a quadratic dependence on the photon energy; the apochromatic case by a cubic dependence, see Fig. 1c. Apochromatic X-ray focusing using lens doublets has been theoretically suggested before^9,10^ but to date has not been experimentally realized.

The practical realization of achromatic and apochromatic X-ray doublets requires adequate refractive and diffractive optics, which has prevented their existence until recently. At higher X-ray energies, efficient diffractive optics require challenging fabrication of high-aspect-ratio structures owing to the low refraction of X-rays in the matter. At lower energies, X-rays are strongly absorbed by matter, and therefore CRL optics become impractical. Moreover, making diverging CRLs requires the fabrication of convex 3D structures: a completely different fabrication route compared to concave converging CRLs that can be made by subtractive fabrication methods such as drilling, punching, or etching. Hence, making precise diverging CRLs with a numerical aperture that matches a high-resolution FZP made by e-beam lithography (EBL) has only recently become possible. At X-ray energies around 7.5 keV, FZP fabrication is well-established for spot sizes down to about 100 nm with high diffraction efficiency. Thanks to the emergence of two-photon polymerization (2PP)-based 3D printing and its evolution into a robust and precise fabrication method for optics^11–22^, fabricating diverging CRLs for X-ray achromats is now feasible. Hence, an achromatic X-ray doublet was realized through the combination of a diverging CRL made by 2PP and an FZP made by EBL. This first X-ray achromatic doublet achieved a sub-micrometer spatial resolution with an achromatic range between 6.0 to 7.2 keV^18^.

**Fig. 2:**
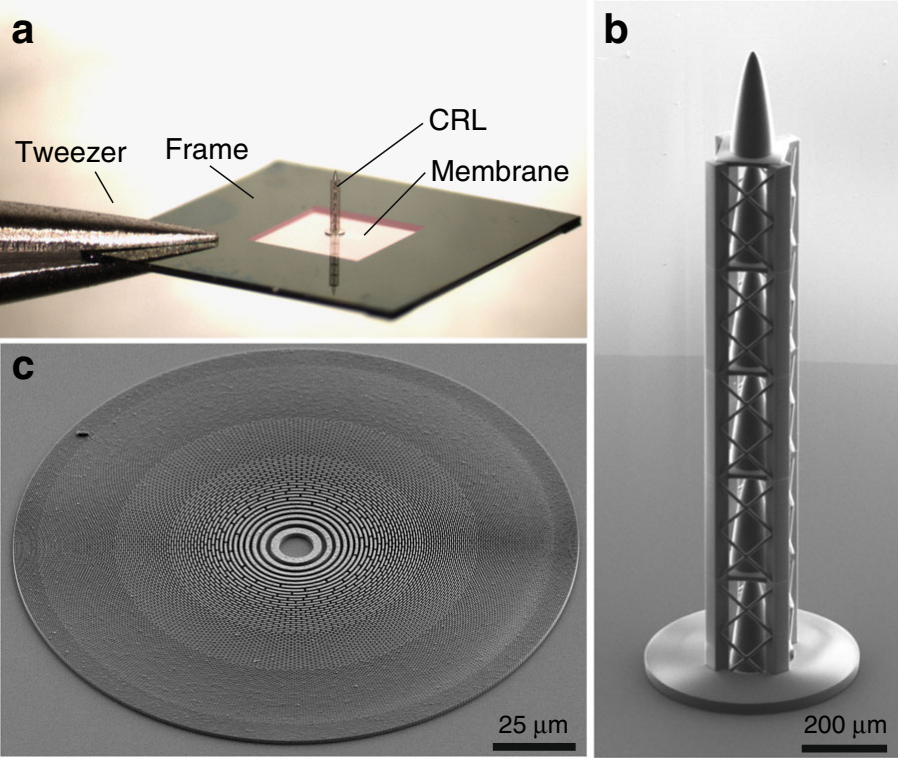
Individual components of the X-ray apochromat. **a** Optical microscope image of the 3D-printed diverging CRL standing on 250-nm thick silicon nitride membrane. The silicon frame is being held from the side by tweezers. **b** Scanning electron microscopy image of the CRL showing the individual refractive lenses with high detail. **c** Scanning electron microscopy image of the FZP under 45-degree tilt angle

In this work, we fabricate an X-ray apochromatic combination and demonstrate its optical performance for the first time. The optical elements composing the X-ray apochromat are shown in Fig. 2. The characterization was based on ptychographic reconstruction of the focal spot and scanning transmission X-ray microscopy (STXM) of a resolution test sample. The apochromat focused X-rays to a spot size of 940 × 740 nm^2^ without significant chromatic aberration over a photon energy range of 7.5 to 12.5 keV.

## Results and discussion

An apochromatic optic was realized as a combination of a diverging CRL fabricated by 2PP-based 3D printing and a converging FZP fabricated via EBL and gold electroplating. Figure 2a shows an overview image of the CRL standing on the 250 nm thick silicon nitride membrane. The scanning electron microscopy image in Fig. 2b shows the excellent surface finish and high-quality fabrication of the 3D-printed CRL. Figure 2c shows a scanning electron microscopy image of the gold FZP. The performance of the apochromat was tested at the P06 beamline of PETRA III of DESY (Hamburg, Germany) in the hard X-ray spectrum. Both STXM and X-ray ptychography measurements were performed at energies between 6.5 and 13.0 keV.

**Fig. 3:**
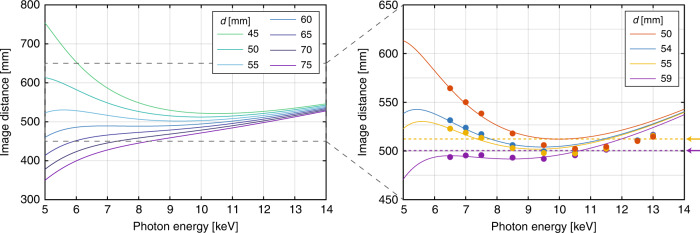
Calculated and experimental image distances for different energies showing the apochromatic behavior. (left) Calculated image distance for the apochromat as a function of photon energy and FZP-CRL separation distance *d*. (right) Experimentally determined image distance for selected *d* (dots) and theoretical predictions (lines). Dotted lines and arrows on the right indicate the sample position for related STXM series in Fig. 4

Changing the separation distance *d* affects the dispersion of the apochromat, see Fig. 3 (left), where theoretical image distance curves are plotted for various photon energies between 5 and 14 keV for seven *d* values. Downstream distances are indicated by positive values. By selecting *d* = 3*f*_d_/8, the linear term of the Taylor series expansion of the image distance is small and the quadratic term is canceled; therefore, dispersion is dominated by the cubic term. For small variations of the *d* value, the curves remain predominantly cubic and a nearly flat dispersion can still be achieved. Thus, *d* can be varied to tune the nature of the chromatic aberration correction. In particular, decreasing *d* shifts the flat dispersion to higher photon energies.

We performed ptychography at separation distances *d* of 50, 54, 55, and 59 mm to measure the *d*-dependent dispersion of the apochromat. The measured image distances, found through the propagation of the reconstructed probe, are shown in Fig. 3 (right) and are in agreement with the theoretical curves, particularly at lower energies. Discrepancies between the theoretical curves and the measured data points in Fig. 3 (right) are seen at higher energies. This difference may be attributed to aberrations in the refractive lens, causing a difference in the CRL’s focal length between its outer and central regions. Because the central part is highly absorbing at low photon energies, this effect would be energy-dependent and could thereby cause the observed discrepancy.

**Fig. 4:**
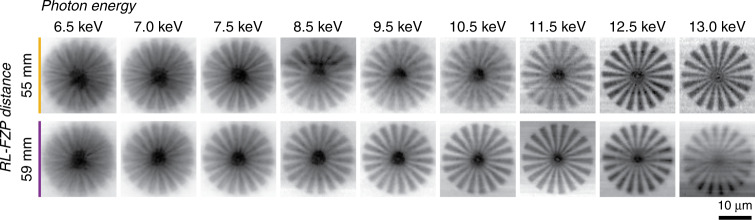
STXM of a Siemens star test sample as a function of photon energy for two FZP-CRL separation distances d. The sample was not moved along the optical axis and was located at the position indicated in Fig. 3

The ptychographic analysis confirms that the dispersion of the apochromat is dominated by a cubic dependence on the energy. To demonstrate apochromatic aberration correction for imaging applications, we performed STXM of a Siemens star test sample at a range of photon energies between 6.5 and 13 keV with both *d* = 55 and 59 mm, as depicted in Fig. 4. Note that for each of the energy scans at a given *d* spacing, the sample position was fixed along the optical axis, indicated by the arrows and dashed lines in Fig. 3 (right). For *d* = 59 mm, i.e., Fig. 4 (bottom row), spokes with the full pitch below 2 µm can be resolved for energies between 7.5 and 12.5 keV. From the STXM image of the Siemens Star test pattern acquired at *d* = 59 mm and *E* = 11.5 keV, the spatial resolution of the apochromat is calculated for a visibility (also known as Michelson contrast) of 0.1 [*I*_max_ − *I*_min_]/[*I*_max_ + *I*_min_]. The estimated half-pitch resolution of the apochromat was 481 nm (see Supplementary Fig. S2). This value matches well with the theoretical expectations. The images at 6.5 and 7.0 keV suffer from aberrations such as blur and ghost images as they lie outside the achromatic range of the apochromat. The intensity distribution of the beam along the optical axis obtained through the propagation of the probe found from ptychography reveal the presence of stronger side lobes at these lower energies. At lower X-ray energies, absorption in the refractive lens is greater thus, the effects of imperfections in the refractive lens are stronger.

The wavefront of the beam at the exit aperture of the lens system was analyzed in order to better understand aberrations, see Supplementary Materials and Fig. S3, where the intensity and phase aberrations are displayed for *d* = 55 mm and a series of photon energies. The phase maps show substantial remaining aberration, particularly at lower photon energies in agreement with the STXM images. Additionally, the size of the beam at the exit aperture decreases with increasing energy due to the weaker refractive power of the CRL. The effects of decreased illumination of the outer edges of the FZP should be further investigated in future studies. Future investigations could also fit wavefront maps at the exit aperture for a bias-free measure of the defocus, though a challenge will be accounting for the energy-dependent absorption of the refractive lens.

**Fig. 5:**
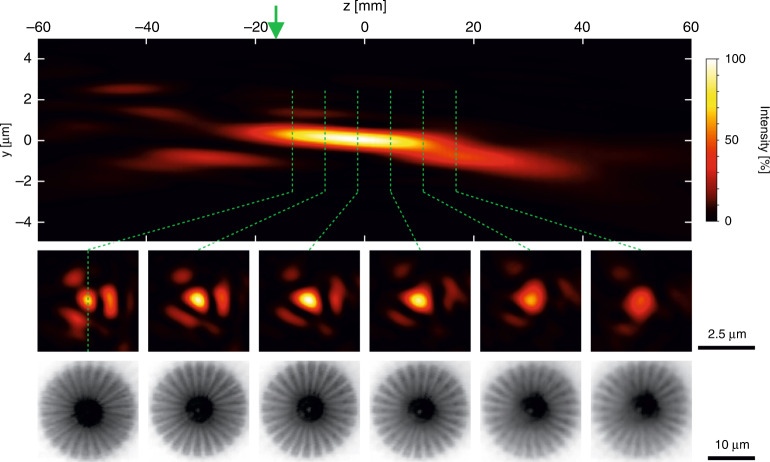
Depth-of-focus of the apochromat. Ptychographic reconstruction and propagation of the probe reveals y − z (top) and x − y (middle) slices through the intensity distribution around the focus. Corresponding STXM scans of a Siemens star test sample are shown for the positions indicated by the dashed green lines (bottom). The green arrow indicates the sample position during ptychographic acquisition

To measure the depth-of-focus and spot size of the apochromat, we performed ptychography at a photon energy of 8.0 keV and separation of *d* = 55 mm. A y-z slice through the propagated probe is shown in Fig. 5 (top row) and x-y slices are shown at several positions along the optical axis (middle row). Depth-of-focus, defined as the z range over which the intensity does not drop below 80% of the overall maximum, was measured to be 18 mm, see Supplementary Fig. S4. This value is greater than for an optic of similar numerical aperture. This result may be attributed to the high absorption of X-rays in the central part of the CRL. It is known that for optics with central obstruction, the depth-of-focus increases significantly^23^. At the plane of maximum intensity, the focus had a full-width-half-maximum size of 940 × 740 nm^2^, see Supplementary Fig. S5. The propagated probe shows agreement with resolution and aberrations in corresponding STXM images of the Siemens Star at a series of positions along the optical axis, as shown in Fig. 5 (bottom row). The significant absorption of the inner part of the CRL implies that the fabrication of the inner part may not be necessary for future developments. In fact, omitting the inner portion of the lens reduces the complexity of the 3D printing fabrication. The refractive lens becomes shorter and it allows for the lenses to be positioned closer together, resulting in a combination that is closer to a thin lens configuration.

## Conclusion

In summary, we have successfully developed an apochromatic X-ray lens system that shows great potential for enhancing X-ray imaging and microscopy. The apochromatic configuration consisted of two independent optical elements: a refractive X-ray lens produced by 2PP-based 3D printing and an FZP fabricated by EBL and gold electroplating. Our apochromat was rigorously characterized at a synchrotron undulator beamline by STXM and ptychographic imaging for photon energies between 6.5 and 12.5 keV. The photon energy-dependent image distance was measured via ptychography and agreed well with the theoretically predicted third-order polynomial dispersion. The apochromat delivered a sub-micron focal spot size capable of resolving 480 nm lines and spaces of a patterned test sample by STXM. While the achieved spatial resolution may appear modest, our results represent a significant advancement due to the four-fold improvement of the apochromatic range (Δ*E*/*E*) over the previously demonstrated achromatic X-ray lens configuration.

In the future, apochromatic X-ray lenses have the potential to become a cost-effective and compact alternative to mirror-based systems, with the additional benefit of being on-axis imaging elements. These lenses would be particularly valuable in time-resolved experiments that demand short exposure time and high signal-to-noise ratios. The spatial resolution of apochromats could be improved by employing optical elements with higher numerical apertures. While the nanofabrication technology is already advanced enough for this push in resolution, an efficient sub-100 nm resolution will be still challenging to realize due to the limiting absorption of the refractive optics. Since X-ray absorption in the matter is significantly reduced for higher X-ray energies, achromatic and apochromatic lens combinations may have much more room for further improvement in the hard X-ray regime. In this respect, fast-evolving developments in 3D printing at the nanoscale will be crucial to enable the fabrication of the required refractive structures. As the development of both achromatic and apochromatic X-ray lenses progresses, we expect them to have an increasingly important impact in the field of X-ray imaging and microscopy and for their scientific applications both in accelerator-based and laboratory X-ray sources.

## Materials and methods

### Design of the apochromatic lens combination

Detailed design considerations and the mathematical derivation for both the achromatic and apochromatic lens combinations for X-ray wavelengths can be found in the literature^10,18^. Briefly, the arrangement of a CRL and an FZP can be interpreted as the combination of two thin lenses. Then, the achromatic condition can be obtained by solving the lens-maker’s equation for the combination of the CRL-FZP in close contact (separation distance *d* = 0) and inserting the dispersion relations for the FZP and CRL, i.e., (Δ*E*/*E*) and (Δ*E*/*E*)^2^, respectively. When the focal length of the CRL is selected such that *f*_r_ = −2*f*_d_, the linear dispersion term in the Taylor series expansion cancels out and the dispersion of the optical system becomes quadratic. The direct implication is that the focal length at two distinct X-ray wavelengths will be the same. This is qualitatively shown in the blue curve of Fig. 1c), with an achromatic range depicted by the arrow at the top of the figure, defined as the range of energy over which the focal length stays within the depth-of-focus. In comparison, the focal length strongly depends on the photon energy for an FZP (red curve) and a CRL (green curve) of comparable focusing power at the design energy of the achromat.

An apochromatic element requires a careful selection of the CRL and FZP focal distances as well as their separation distance to minimize or completely eliminate the linear and quadratic dispersion terms. This is achieved for the condition *f*_r_ = −9*f*_d_/8 and a separation distance of *d* = 3*f*_d_/8. The resulting image distance of the apochromat is *l*_apo_ = 3*f*_d_ measured from the FZP element. This is depicted schematically in Fig. 1b, and the resulting dispersion and depth-of-focus are shown by the yellow curve in Fig. 1c. The range of the chromatic aberration correction is qualitatively shown by the arrow at the top of the plot in Fig. 1c. A further geometrical optics consideration of the apochromat as a combination of two thin lenses can be found in the supplementary material (Fig. S1). To achieve the same focal spot size as in the achromatic case, the apochromatic embodiment is much more challenging to fabricate. In an apochromatic design, the CRL must have ∼2.66 times greater defocusing power. Hence, the CRL needs to be 2.66 times taller. Taller lenses are more sensitive to tilt errors, so the lens axis needs to be aligned with better precision with respect to the optical axis. The diffractive element in the apochromatic condition is also more challenging, requiring an FZP with 1.5 times greater focusing power to achieve a similar focal spot size. In both achromatic and apochromatic designs, the sequential order of the diffractive and refractive optics plays a role. Recently, ref. ^10^ have referred to achromatic and apochromatic combinations where the refractive lens is upstream and the diffractive lens is downstream (embodiments represented in Fig. 1a, b) as type I doublets and the reverse sequence as type II. The type I apochromats have greater focusing power for all *d* values^10^.

In the case of apochromatic design, the separation distance *d* between the refractive and diffractive elements has to be taken into consideration. For type I apochromats where the diverging refractive lens is upstream, the X-rays will diverge until they reach the FZP. Hence, the FZP needs to be larger in diameter than the CRL to collect this divergent beam. Since the divergence is energy dependent, the diameter of the FZP is calculated considering the lowest energy (most divergent) X-rays that the apochromat is planned to focus.

In our realization, the apochromat was designed for a central energy of 7.5 keV and for a focal spot size of about 730 nm. The diameter of the refractive lens was selected to be 100 µm. This aperture size is a good compromise between the light collection and the required CRL height. Much smaller aperture sizes result in a low amount of light collection, making the optic impractical to use, and much larger aperture sizes require the refractive lens to be very tall, more challenging to fabricate, and cause significantly more X-ray absorption. To achieve the 730 nm focal spot size, the refractive lens needs to be 1455 µm tall, giving a radius of curvature of 0.86 µm. In practice, the refractive lens was divided into 6 refraction surfaces, each 242.5 µm tall (see Fig. 2b), to reduce the complexity of the 2PP-based 3D printing. The matching FZP at the design energy has a diameter of 133 µm and an outermost zone width of 200 nm. However, to accommodate the lower energy end of the apochromat range, which in this case was about 5.5 keV, the FZP was extended to a diameter of 161.6 µm and an outermost zone width of 166 nm.

### Fabrication of the apochromatic lens combination

The apochromatic lens combination used in our experiment consisted of a converging Fresnel zone plate and a diverging compound refractive lens. The two elements were fabricated on two separate silicon nitride membranes. The FZP was fabricated using EBL, followed by gold electroplating. First, the silicon nitride membrane substrate was coated with chromium-gold-chromium layers by thermal evaporation. Then, a 1.6 µm thick PMMA resist layer was spin-coated. The FZP was patterned into the PMMA resist by Raith EBPG 5000+ EBL instrument (Raith GmbH, Germany) at 100 kV electron acceleration voltage. After developing in an isopropanol-water mixture, the FZP structures were formed with gold electroplating and the PMMA residual was removed with acetone^24^.

The CRL was designed using free CAD software, OpenSCAD. The diverging CRL was fabricated using a commercial 2PP-based 3D printing instrument, Photonic Professional GT+ (Nanoscribe, Germany). A silicon nitride membrane with a thickness of 250 nm was used as a substrate. A commercial acrylate-based negative photoresist (IP-S, Nanoscribe, Germany) was used in dip-in lithography mode with a 25× objective. The CRL was composed of six identical stacked lenses. Each individual lens had an aperture of 100 µm and a height of 242.5 µm to match the design parameters of the FZP. The X-ray absorption and diffraction properties of the CRL were estimated assuming a chemical formula C_14_ H_18_ O_7_ and a volumetric mass density of 1.2 g cm^−3^.

### Characterization of the apochromatic lens combination

The apochromat was characterized at the beamline P06 of the PETRA III storage ring of DESY (Hamburg, Germany)^25^. Here, a Si (111) double-crystal monochromator was used to obtain monochromatic X-rays with photon energies between 6.5 and 13.0 keV (Δ*E*/*E* ≈10^−4^). The apochromat was located ~93 m from the undulator source.

The X-ray beam size was matched to the aperture of the apochromat with an upstream pinhole of 100 µm diameter. The CRL was mounted upstream of the FZP with variable separation distance. The CRL was mounted on a motorized stage to allow for angular alignment of the ∼1.6-mm-tall CRL with the optical axis. Higher diffraction orders were removed by an order-sorting aperture with a 30 µm diameter placed about 20 mm before the sample. A Siemens star pattern made of gold was mounted as a sample on a translation stage ~500 mm downstream of the FZP. The Siemens star had a diameter of 20 µm and consisted of 18 spokes.

Scanning transmission X-ray microscopy (STXM) and ptychographic X-ray imaging were used to characterize the performance of the apochromatic lens combination. STXM is a microscopy technique where images of the sample under investigation are acquired by raster-scanning a focused X-ray beam across the sample while measuring the absorption, scattering, or fluorescence signals^26^. STXM can obtain images with structural, elemental, and chemical properties of the sample at a high spatial resolution down to the nanometer scale, limited by the size of the focal spot. On the other hand, X-ray ptychographic imaging^27^ is an advanced imaging technique that employs a coherent X-ray beam to obtain an image of the sample. This method involves illuminating overlapping regions of the sample while collecting the diffraction patterns with a pixelated detector. The collected diffraction patterns are then used to reconstruct a high-resolution amplitude and phase image of the sample using computational iterative algorithms. In addition to the sample information, the ptychographic reconstruction obtains the amplitude and phase of the wavefield used to probe the sample. As a result, this technique has been demonstrated as a powerful tool for the characterization of X-ray optical elements. For both STXM and ptychography measurements, an Eiger 4 M detector (2 × 4 modules, each with 2070 × 2167 array of 75 × 75 µm^2^ pixels, manufactured by Dectris AG, Switzerland) located 8.4 m downstream of the sample was used.

STXM scans were acquired with a continuous raster-scan of a 24 × 24 µm^2^ grid with a step size of 0.3 µm. Exposure time per pixel was varied from 100 to 200 ms in order to match the beam intensity variations at each photon energy. The X-ray transmission signal at every raster-scan position was obtained by integrating the intensity within the cone of the direct beam of the acquired diffraction pattern^28^. Intensities were normalized using an upstream quadrant beam position monitor. For display purposes, the STXM images were cropped to 72 × 72 pixels about the center of the Siemens star test sample and intensities were mapped from [*I*_min_, *I*_max_] → [0, 255].

Ptychography measurements were performed by raster-scanning a 16 × 16 µm^2^ grid with a step size of 0.5 µm. The exposure time per position was adjusted from 100−200 ms in order to match the beam intensity changes for each photon energy. Ptychographic reconstruction was performed with an in-house algorithm based on work from Maiden and Rodenburg^29^.

## Supplementary information

Supplementary Material
