# Supplementary material for: Apochromatic X-ray focusing
Source: Light Sci Appl. 2023 May 4;12:107. doi: 10.1038/s41377-023-01157-8 (PMC10160054; doi:10.1038/s41377-023-01157-8)
Supplement: Supplementary file 1 — Supplementary Material [file 41377_2023_1157_MOESM1_ESM.docx]

Supplementary Information

for

Apochromatic X-ray focusing

UMUT T. SANLI1,*,†, GRIFFIN RODGERS2,†, MARIE-CHRISTINE ZDORA1, PENG QI1, JAN GARREVOET3, KEN VIDAR FALCH3, BERT MÜLLER2, CHRISTIAN DAVID1, JOAN VILA-COMAMALA1

*1Paul Scherrer Institute, Laboratory for X-ray Nanoscience and Technologies, Forschungsstrasse 111, 5232 Villigen, Switzerland*

*2Biomaterials Science Center, Department of Biomedical Engineering, University of Basel, Hegenheimermattweg 167 B, 4123 Allschwil, Switzerland*

*3 Deutsches Elektronen-Synchrotron DESY, Notkestr. 85, 22607 Hamburg, Germany*

**Corresponding author:* [*umut.sanli@psi.ch*](mailto:umut.sanli@psi.ch)

†*Equal contribution*

# Supplementary Information

- 1. *Description of the Apochromatic Lens Configuration*

The apochromatic lens configuration can be described as combination of two thin lenses located at 𝐴_1_ and 𝐴_2_ separated by a distance 𝑑 = 3/8 𝑓_𝑑_, as depicted in figure [S1](file:///C:\apochromat%20paper\revised%20version%20word%20documents\An_Apochromatic_X_ray_Optic__Revised_for_MS%20Word_.docx#_bookmark22)a). The first thin-lens is diverging with a focal length 𝑓_1_ = 𝑓_𝑟_ = −9/8 𝑓_𝑑_ while the second one is converging with a focal length 𝑓_2_ = 𝑓_𝑑_. Following an Optics textbook such as [[1](file:///C:\apochromat%20paper\revised%20version%20word%20documents\An_Apochromatic_X_ray_Optic__Revised_for_MS%20Word_.docx#_bookmark21)], the resulting lens combination can be described by means of a resulting focal length 1/ 𝑓 = 1/ 𝑓_1_ + 1/ 𝑓_2_ − 𝑑/( 𝑓_1_ 𝑓_2_) = 4/(9 𝑓_𝑑_) and the principal planes 𝑈_𝑜_ and 𝑈_𝑖_ located at 𝐴_1_𝑈_𝑜_ = 𝑓 𝑑/ 𝑓_2_ = 27 𝑓_𝑑_/32 and 𝐴_2_𝑈_𝑖_ = − 𝑓 𝑑/ 𝑓_1_ = 3 𝑓_𝑑_/4, respectively. As shown in figure [S1](file:///C:\apochromat%20paper\revised%20version%20word%20documents\An_Apochromatic_X_ray_Optic__Revised_for_MS%20Word_.docx#_bookmark22)a), the focal length 𝑓 is measured from the principal planes

at both object and images spaces. A point source placed at infinity will be focused at F, which corresponds to an image distance 𝑙_𝑖_ = 𝐴_2_𝐹 = 𝑓 + 𝐴_2_𝑈_𝑖_ = 3 𝑓_𝑑_, measured from the second lens element. By introducing the energy dependence of the focal length of the refractive and diffractive lenses for the particular values of the realized X-ray apochromat, Figure [S1](file:///C:\apochromat%20paper\revised%20version%20word%20documents\An_Apochromatic_X_ray_Optic__Revised_for_MS%20Word_.docx#_bookmark22)b) shows the energy dependence of the focal length, the principle plane 𝑈_𝑖_ and image distance 𝑙_𝑖_ of the

apochromatic lens combination. Notice that only the image distance follows the expected cubic functional behaviour while the resulting focal length still changes with the energy. As a result, if such lens configuration would be used for imaging, the magnification would still vary with the energy, thus suffering from lateral chromatic aberration.


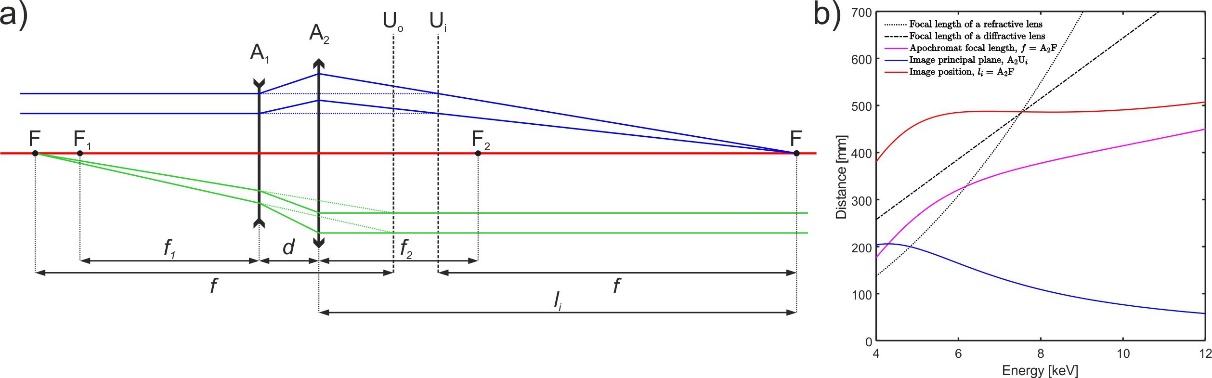


Fig. S1. a) The apochromatic lens configuration can be described as combination of two thin lenses located at 𝐴_1_ and 𝐴_2_ and separated by a distance 𝑑. The resulting configuration can be described in terms of the apochromatic focal length 𝑓 = 9 𝑓𝑑 /4 and the principal planes 𝑈𝑜 and 𝑈i. A point source at infinity will be focused at an image distance 𝑙𝑖 = 3 𝑓𝑑 from the second lens located at 𝐴_2_. b) The plot shows the focal length, principal planes and the image distance of the apochromat as function of

photon energy for the particular values of the realized configuration. Notice that only the image distance of the apochromat follows the expected cubic function behaviour. The focal lengths of a FZP and a refractive lens of comparable focusing power at the design energy are shown for comparison.

- 1. *Wavefield at the Exit Aperture of the Apochromat*

In addition to the intensity distribution in the vicinity of the focus, the wavefield was analyzed at the exit aperture of the lens system, i.e. at the position of the FZP. To achieve this, the beam was propagated in 250 µm steps ± 20 mm from the object plane. The highest intensity peak was identified and used as the probe position. The probe was then propagated to the aperture plane. To avoid interference artefacts in the far-field, a circular mask was applied to the probe before

propagation to the aperture. The phase was evaluated in the aperture plane, and the phase of a spherical wave originating from the probe position was subtracted before unwrapping. The


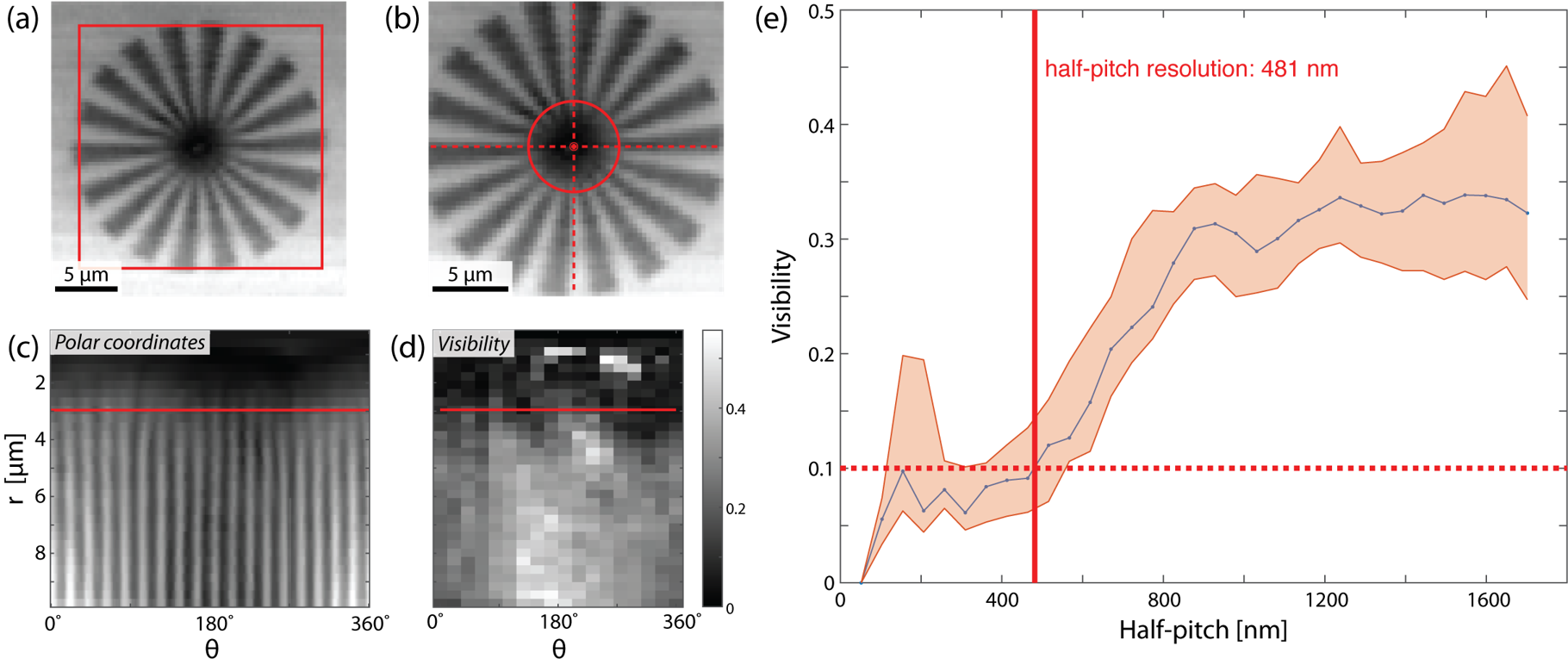


Fig. S2. Spatial resolution measured from a STXM image of a Siemens star test pattern for the apochromat with d = 59 mm and E = 11.5 keV. The orignal STXM image

(a) was cropped and centered (b), then converted to polar coordinates (c). Visibility, [𝐼𝑚𝑎𝑥 − 𝐼𝑚𝑖𝑛]/[𝐼𝑚𝑎𝑥 + 𝐼𝑚𝑖𝑛] was calculated as a function of radius (d). The median (25𝑡ℎ, 75𝑡ℎ percentile) of the visibility are plotted as a function of the test pattern

half-pitch (e). Half-pitch resolution is based on a median visibility threshold of 0.1, with solid red lines indicating this cut-off in (b-e).

unwrapping was done by the unwrap_phase function in the *skimage* Python module.

The resulting intensity and phase aberrations are displayed in Figure [S3](file:///C:\apochromat%20paper\revised%20version%20word%20documents\An_Apochromatic_X_ray_Optic__Revised_for_MS%20Word_.docx#_bookmark24) for a spacing of 𝑑 = 55 mm across a series of photon energies. The root-mean-square (RMS) of the phase aberrations are displayed. Note that for all photon energies, the aberrations are larger than the Maréchal criteria for a diffraction limited source, which is 1/14 = 0.072 waves RMS or 0.449 radians. The aberrations are strongest for lower photon energies in good agreement with STXM measurements.


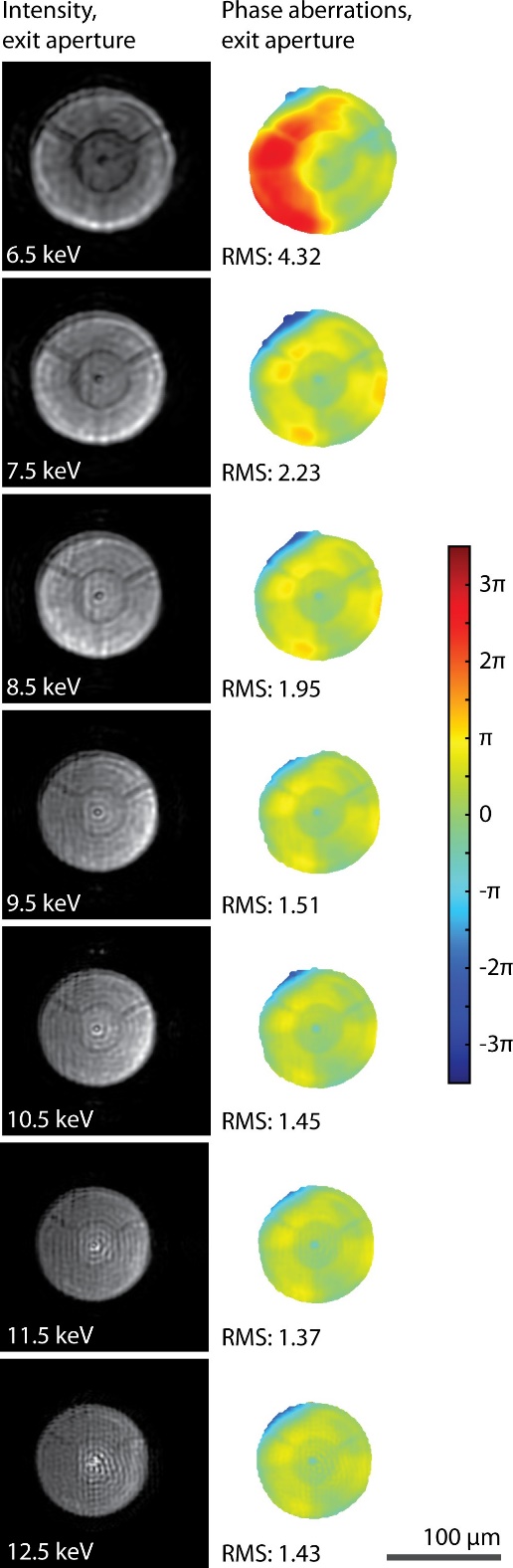


Fig. S3. Intensity (left) and phase aberrations (right) of the wavefront at the exit aperture of the lens for a spacing of d = 55 mm across a series of photon energies. The root-mean-square (RMS) of the phase aberrations within the masked regions are shown.


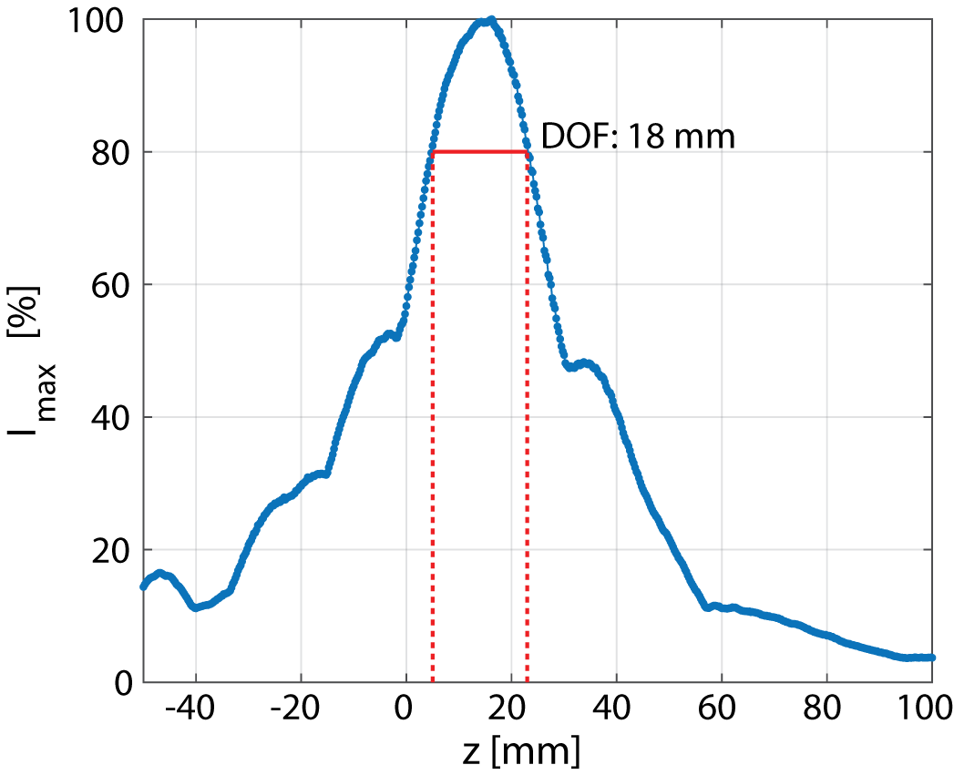


Fig. S4. Depth-of-focus for the apochromat with d = 55 mm at E = 8 keV. Ptychographic reconstruction of the probe and propagation revealed the beam caustics. The maximum intensity of each x-y plane is plotted, normalized by the overall maximum intensity. A depth-of-focus of 18 mm was calculated based on the range of z over which the intensity is greater than or equal to 80% of the overall maximum intensity.


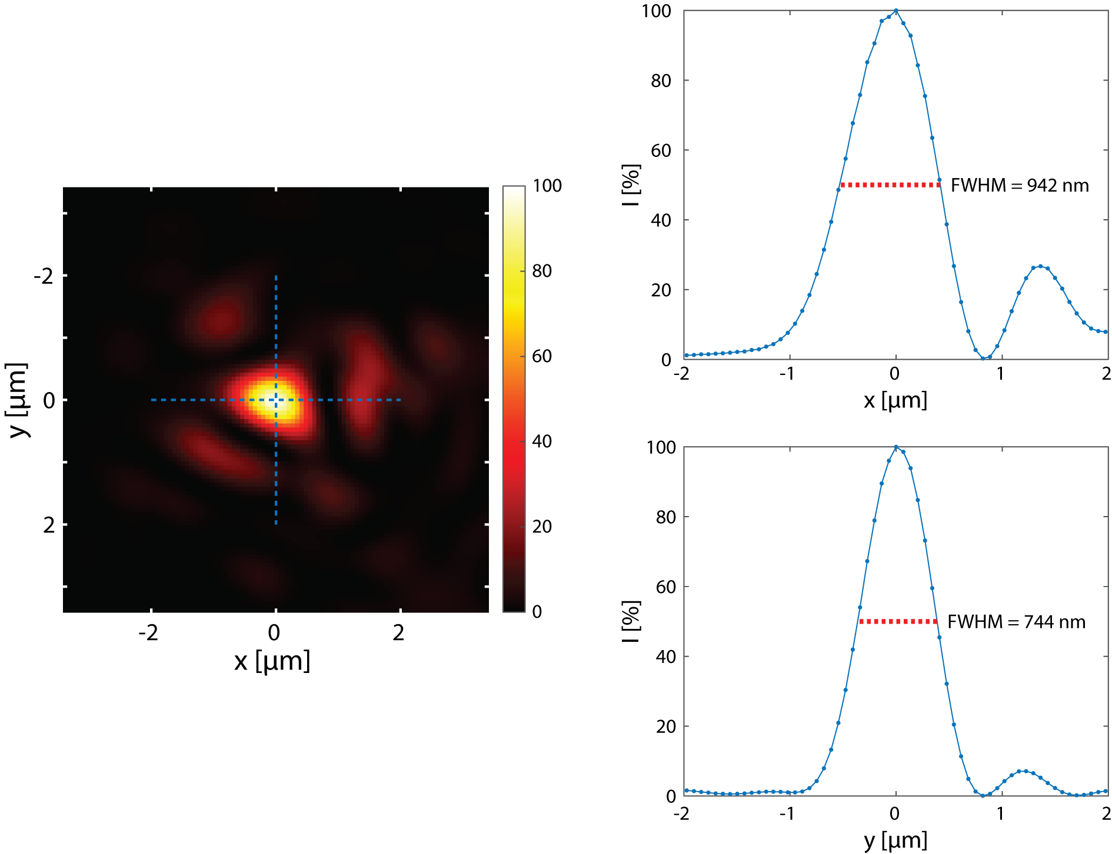


Fig. S5. Spot size for the apochromat with d = 55 mm at E = 8 keV. Ptychographic reconstruction of the probe and propagation revealed the intensity distribution of the beam. The x-y plane of the focus is shown (left), as defined by the plane where overall maximum intensity is found. Line profiles in the x- and y-directions are shown, with the related full-width-half-maximum (FWHM) shown (right top and bottom, respectively). The mean FWHM spot size was 843 nm.

Reference:

[1] F. Jenkins and H. White, *Fundamentals of Optics*, McGraw-Hill education books (McGraw-Hill, 2018).
